# T1 measurements in a rat model of acute myocardial ischemia/reperfusion

**DOI:** 10.1186/1532-429X-18-S1-Q18

**Published:** 2016-01-27

**Authors:** Darach O h-Ici, Sarah Jeuthe, Titus Kuehne, Felix Berger, Sebastian Kozerke, Daniel Messroghli

**Affiliations:** 1 grid.418209.60000000100000404German Heart Institute, Berlin, Germany; 2 grid.5801.c0000000121562780University and ETH Zurich, Zurich, Switzerland

## Background

Myocardial ischemia causes local edema, leading to prolongation of both T1 and T2. In clinical MRI, T2-weighted techniques are commonly used to visualize myocardial edema. The aim was to study the acute T_1_ changes in-vivo in a novel closed chest animal model of myocardial ischemia/reperfusion using T1 mapping by SALLI in order to test if this parametric approach could provide additional diagnostic information as compared to conventional MRI.

## Methods

5 groups of rats had an inflatable balloon coronary occluder surgically inserted via thoracotomy. They were allowed to recover for 7 days. MRI was performed to obtain baseline measurement of ventricular function. T_1_ mapping was performed using the Small-Animal Look-Locker Inversion Recovery (SALLI) technique. Short axis SALLI MR imaging was performed using the same short axis orientation in the mid ventricle distal to the occluder, with SALLI parameters as previously described. Without removing the animals from the scanner, the left coronary artery was occluded for 15, 30 or 60 minutes. 2 groups of animals underwent 3 cycles of 5 minutes of preconditioning before 30 and 60 minutes of ischemia. Myocardial T_1_ was measured was repeated at 15 minutes intervals during the experiment, throughout ischemia and the 90 minutes of reperfusion. MRI was performed on a whole-body 3.0-T MR unit with a dedicated rat coil.

## Results

Myocardial T_1_ increased in the area-at-risk (AAR) within the first 15 minutes of ischemia. T_1_ values increased slightly with longer periods of ischemia, but did not change with myocardial reperfusion. Preconditioning led to a more gradual and lesser increase in myocardial T_1_ in the AAR. Changes in T_1_ persisted at 3 and 7 days.

## Conclusions

Myocardial T1 mapping is able to study the changes in myocardial T_1_ in real time in a model of ischemia/reperfusion. Changes in myocardial T_1_, likely reflecting myocardial water content, occur early following the onset of myocardial ischemia and values remain elevated for at least seven days following ischemia. Preconditioning appears to lead to less myocardial oedema, which may explain some of its protective effects in myocardial ischemia. T1 mapping might provide an imaging marker for monitoring the quality of myocardial reperfusion.

**Figure 1:**
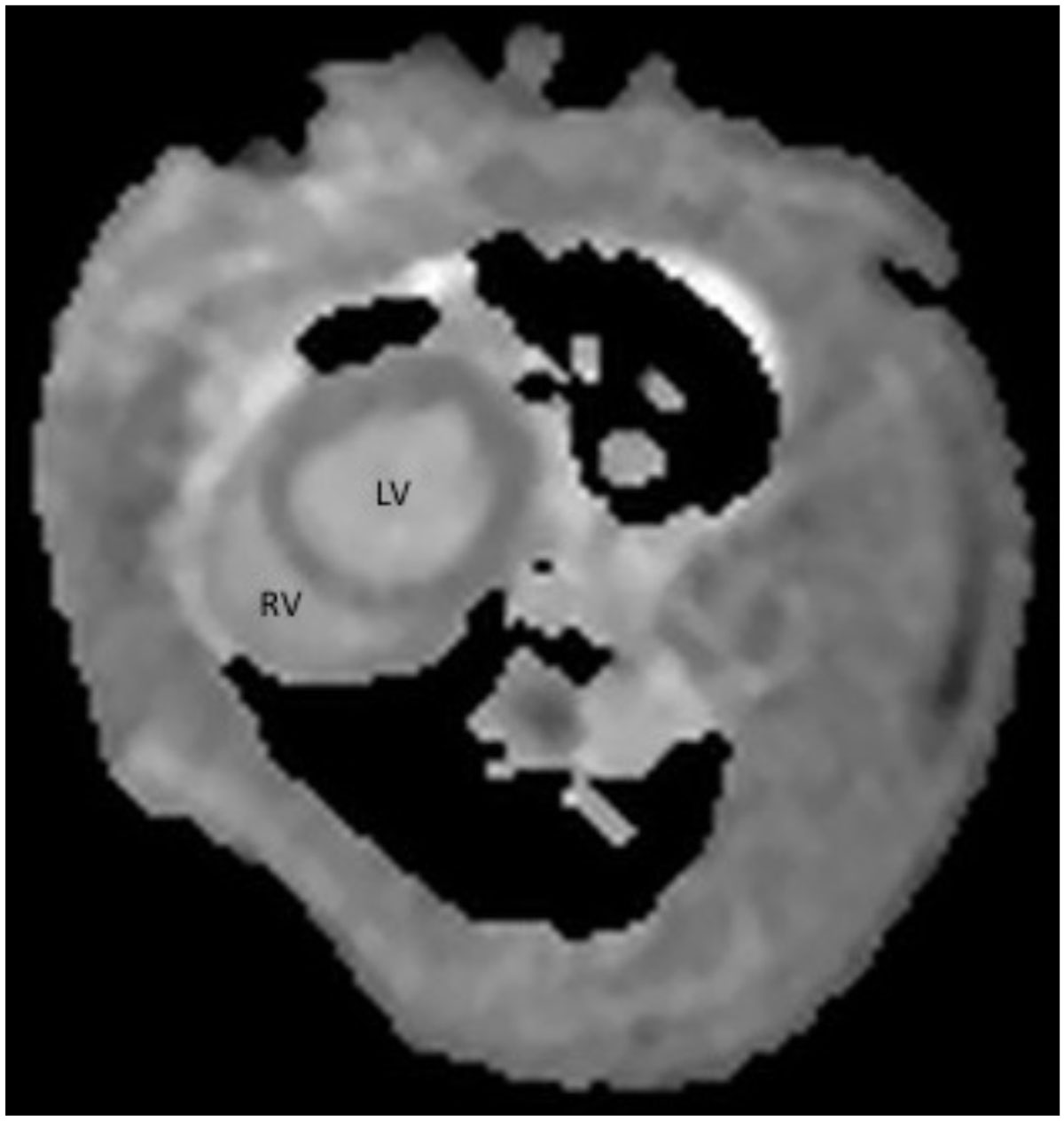
**Short axis T**_**1**_ **map of the left ventricle (LV) prior to myocardial ischemia demonstrating homogenous signal across the myocardium at baseline**. The walls of the right ventricle (RV) may also be identified.

**Figure 2:**
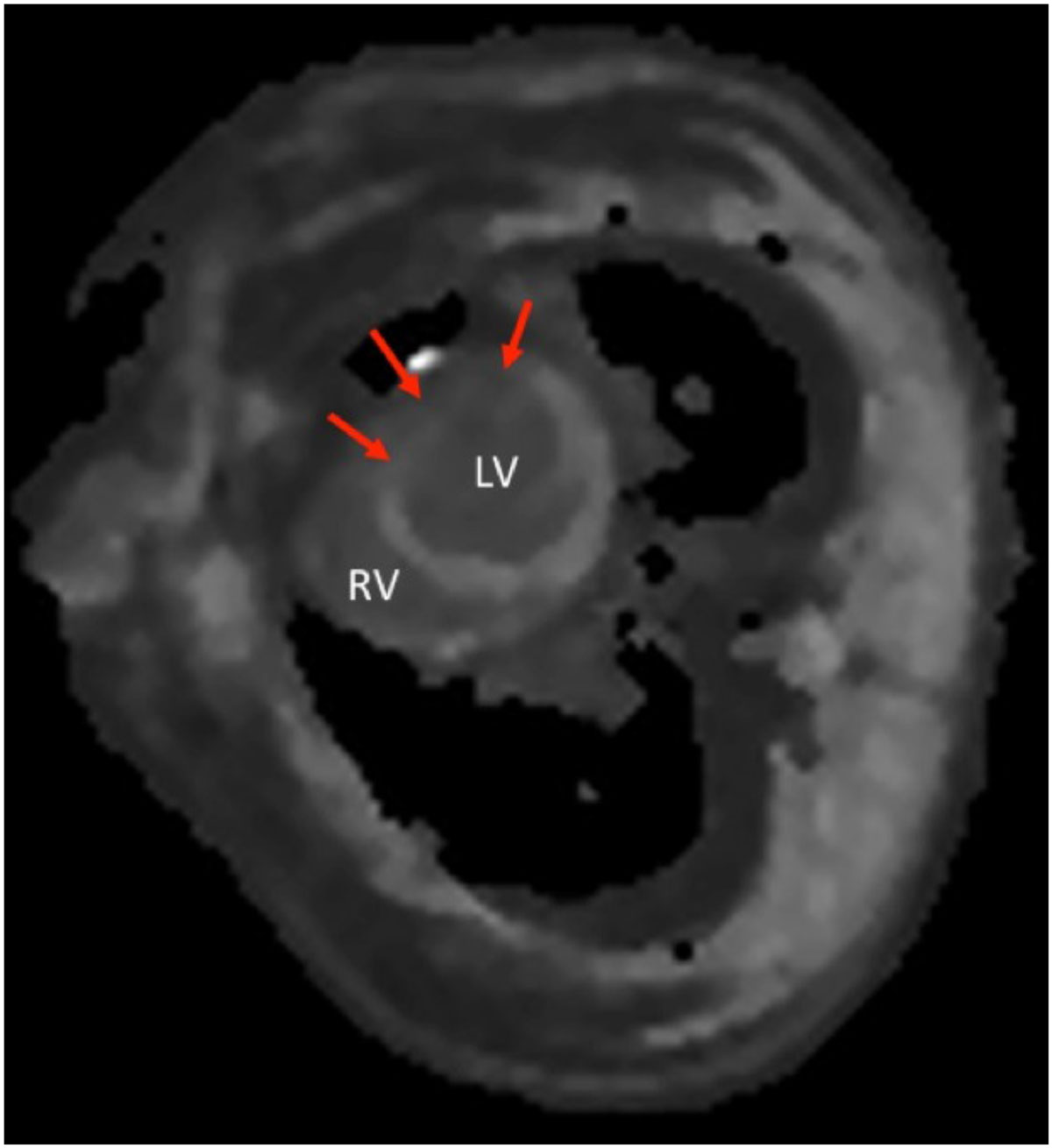
**Post contrast T**_**1**_ **map demonstrating a large anterior segment of myocardial injury (arrows) in the left ventricle following 60 minutes of myocardial ischemia**.

